# Supplementary material for: eQTL Catalogue 2023: New datasets, X chromosome QTLs, and improved detection and visualisation of transcript-level QTLs
Source: PLoS Genet. 2023 Sep 18;19(9):e1010932. doi: 10.1371/journal.pgen.1010932 (PMC10538656; doi:10.1371/journal.pgen.1010932)
Supplement: S2 Text — (PDF) [file pgen.1010932.s002.pdf]

Classification criteria for QTL coverage plots

Examples of primary splicing QTLs

UKBB.VitD_chr11_11577947-18159734

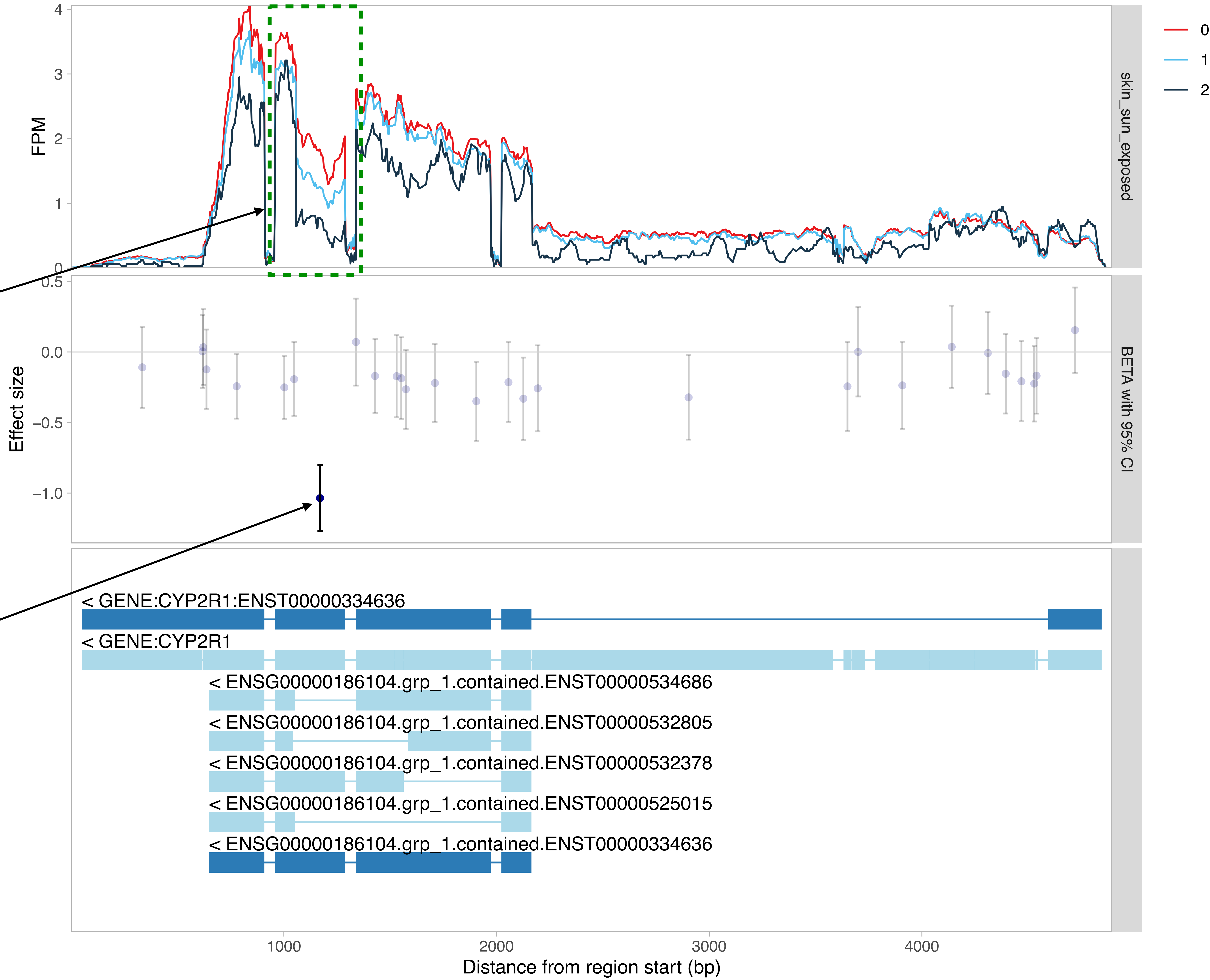

Visible genotype-dependent difference read coverage across the affected exon relative to neighbouring exons.

Exon-level QTL signal markedly stronger for one exonic part relative to other exons of the gene.

Decision: primary sQTL

UKBB.VitD_chr16_80499957-83500099

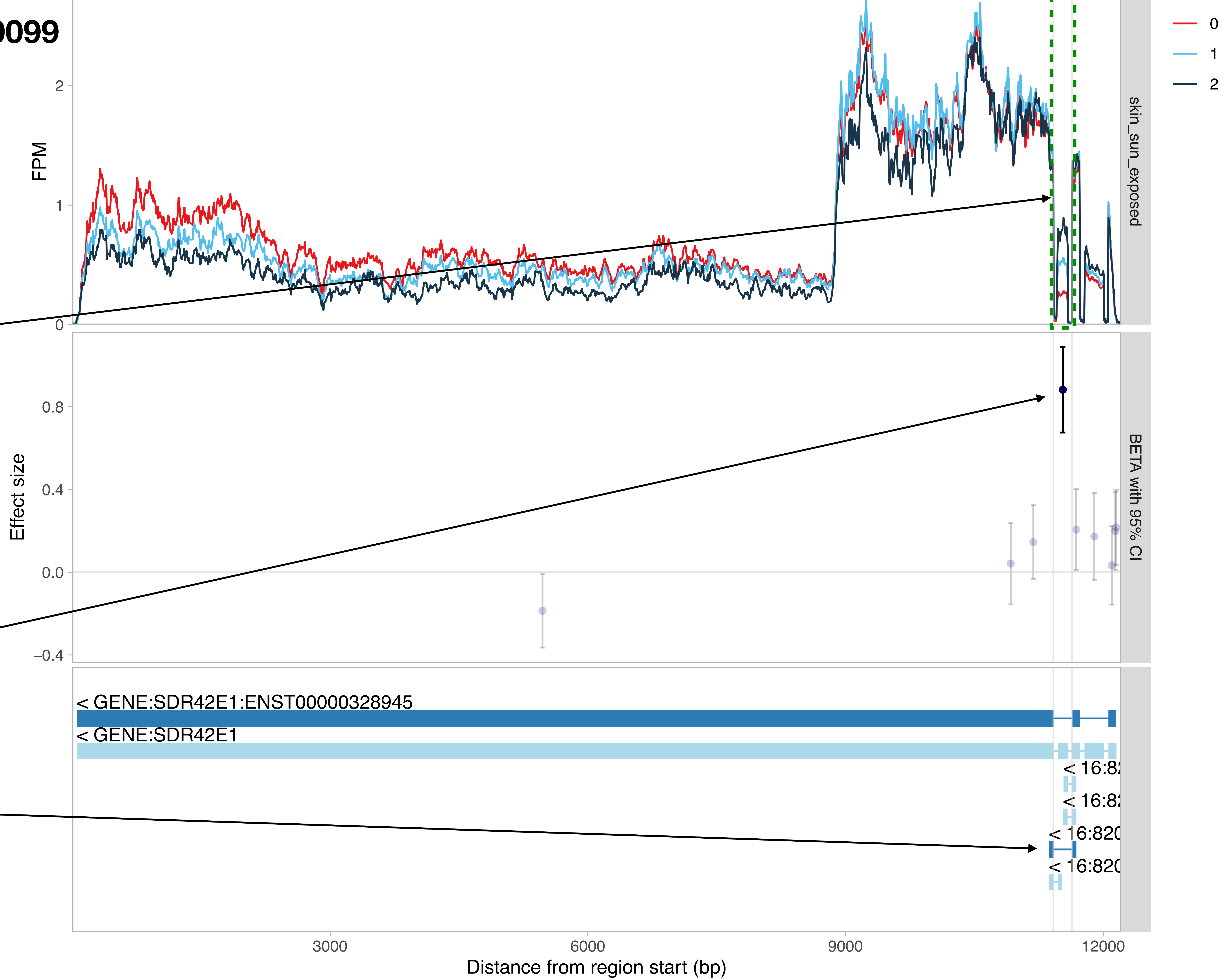

Visible genotype-dependent difference in read coverage across the affected exon relative to neighbouring exons.

Exon-level QTL signal markedly stronger for one exon relative to other exons of the gene.

Most strongly associated Leafcutter cluster matches the affected exon.

Decision: primary sQTL

UKBB.VitD_chr16_80499957-83500099

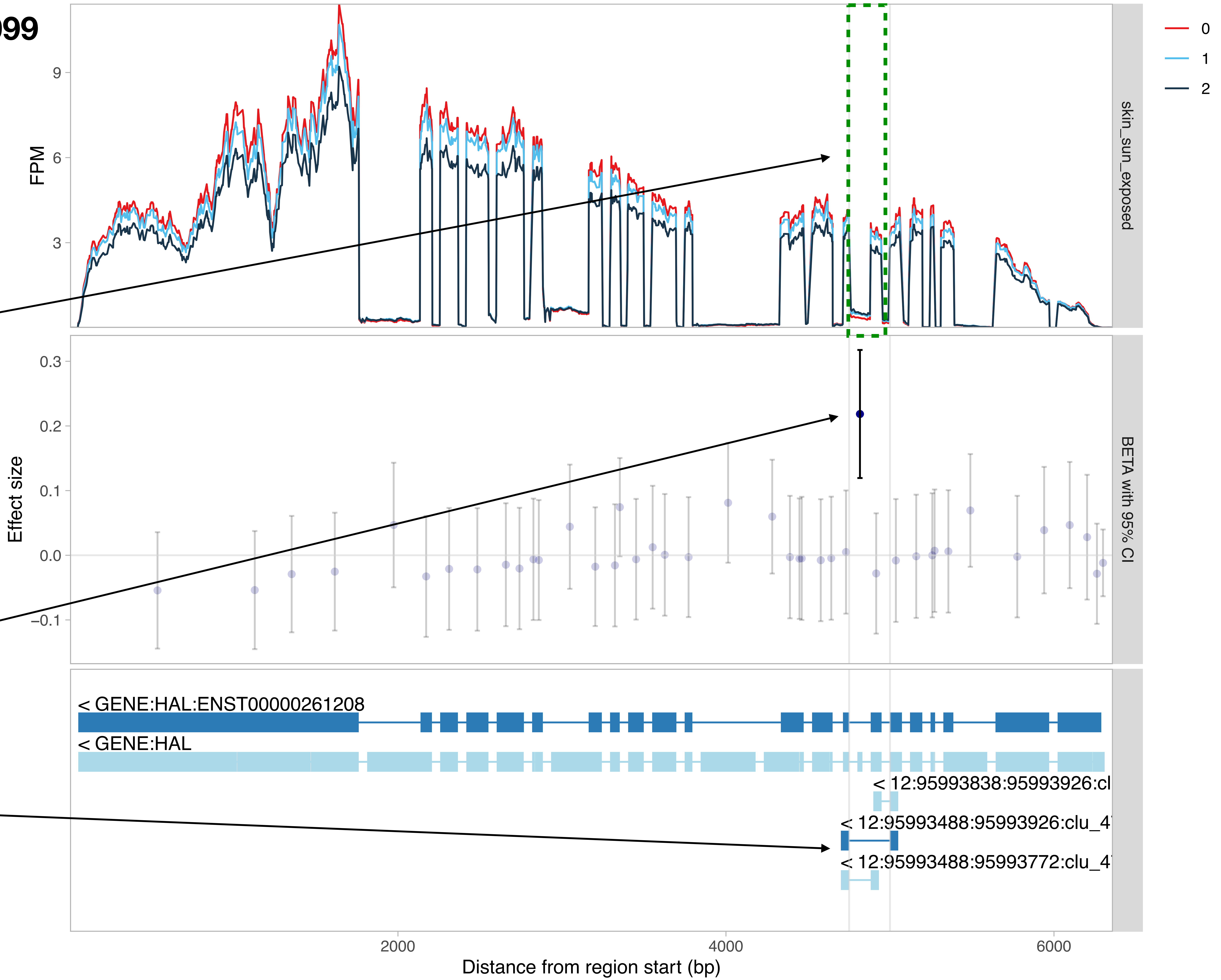

The read coverage signal is noisy, but suggests potential intron retention event or noisy splicing.

Exon-level QTL signal markedly stronger for one exon.

Most strongly associated Leafcutter junction skips the affected exon.

Decision: primary sQTL

Examples of primary expression QTLs driving secondary splicing QTL signals

UKBB.VitD_chr1_107774781-110774507

Large genotype-dependent differences across all exons indicates a strong eQTL signal.

Most strongly associated Leafcutter junction has weak support in the read coverage and exon-level tracks, suggesting that it represents a low frequency splice junction.

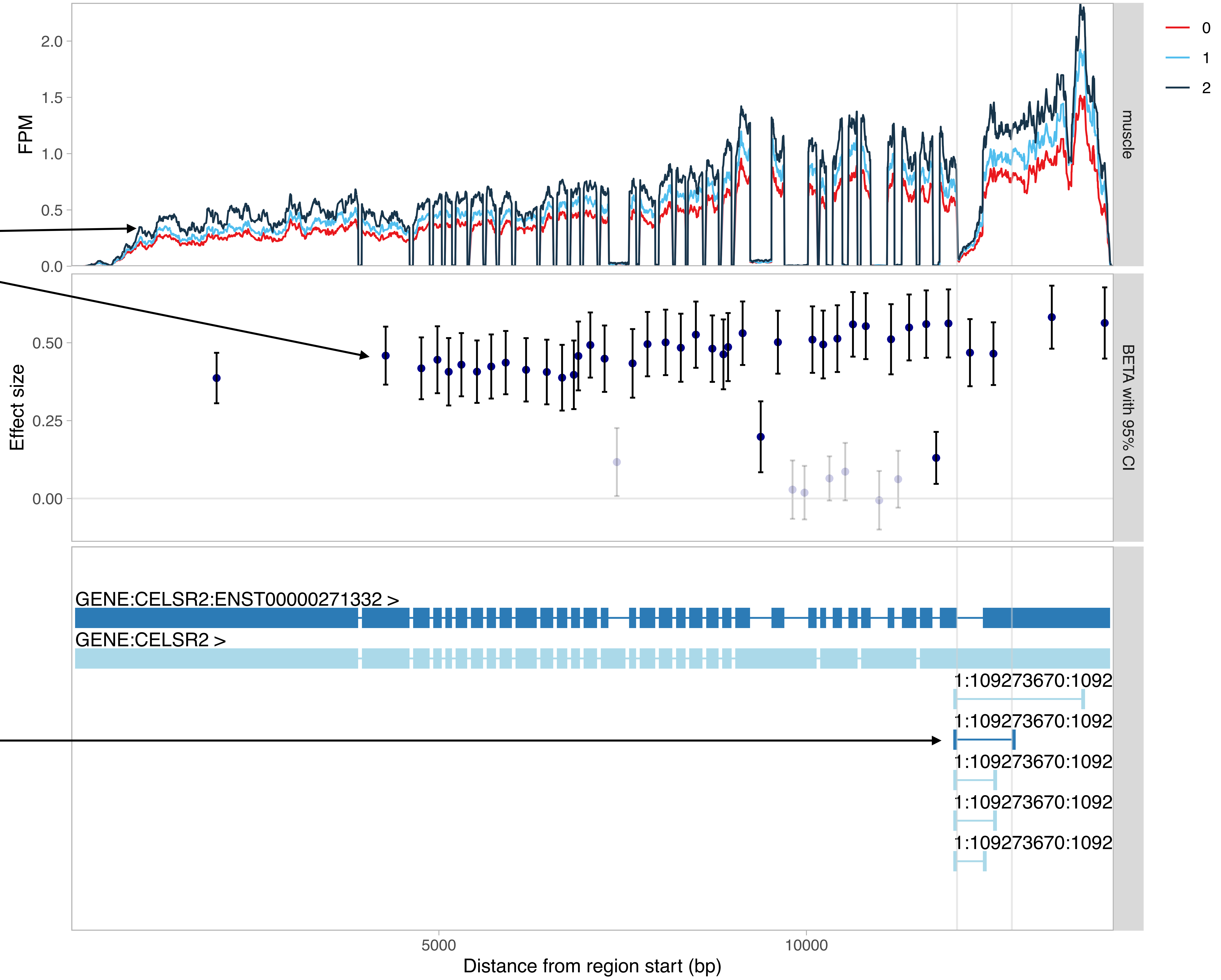

Decision: likely primary eQTL that manifests as a secondary sQTL

UKBB.VitD_chr4_67577400-75046787

Large genotype-dependent differences across all exons indicates a strong eQTL signal.

Dip in read coverage near the affected exon-exon junctions suggest that there might be a region with low mappability.

Exon skipped by the most strongly associated Leafcutter junction has exactly the same exon-level effect size as neighbouring exons.

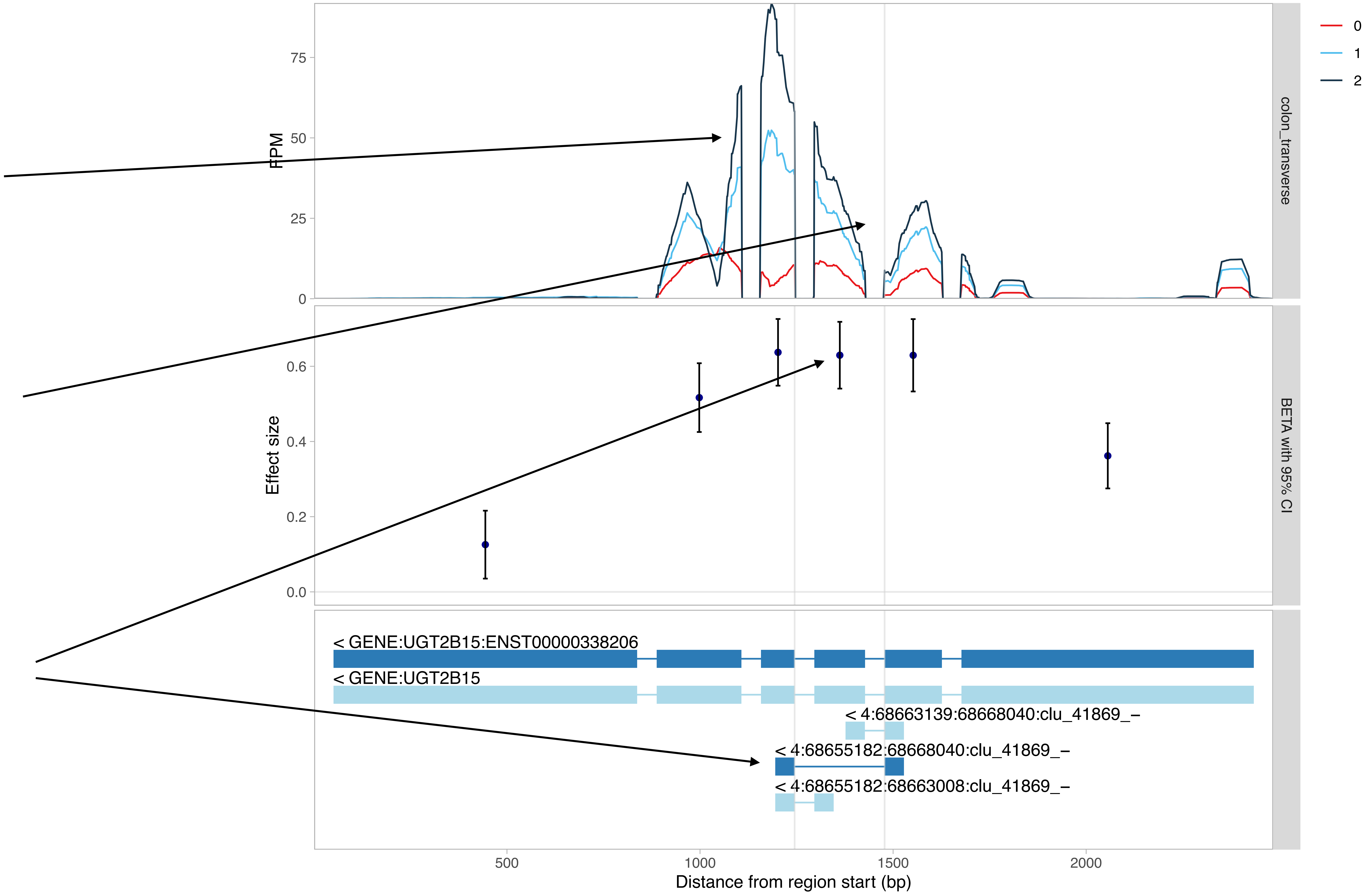

Decision: likely primary eQTL that manifests as a secondary sQTL or reference mapping bias

UKBB.VitD_chr4_85880379-88880005

Large genotype-dependent differences across all exons indicates a strong eQTL signal.

Affected exon is very lowly expressed relative to neighbouring exons.

Most strongly associated Leafcutter junction skips the lowly expressed alternative exon.

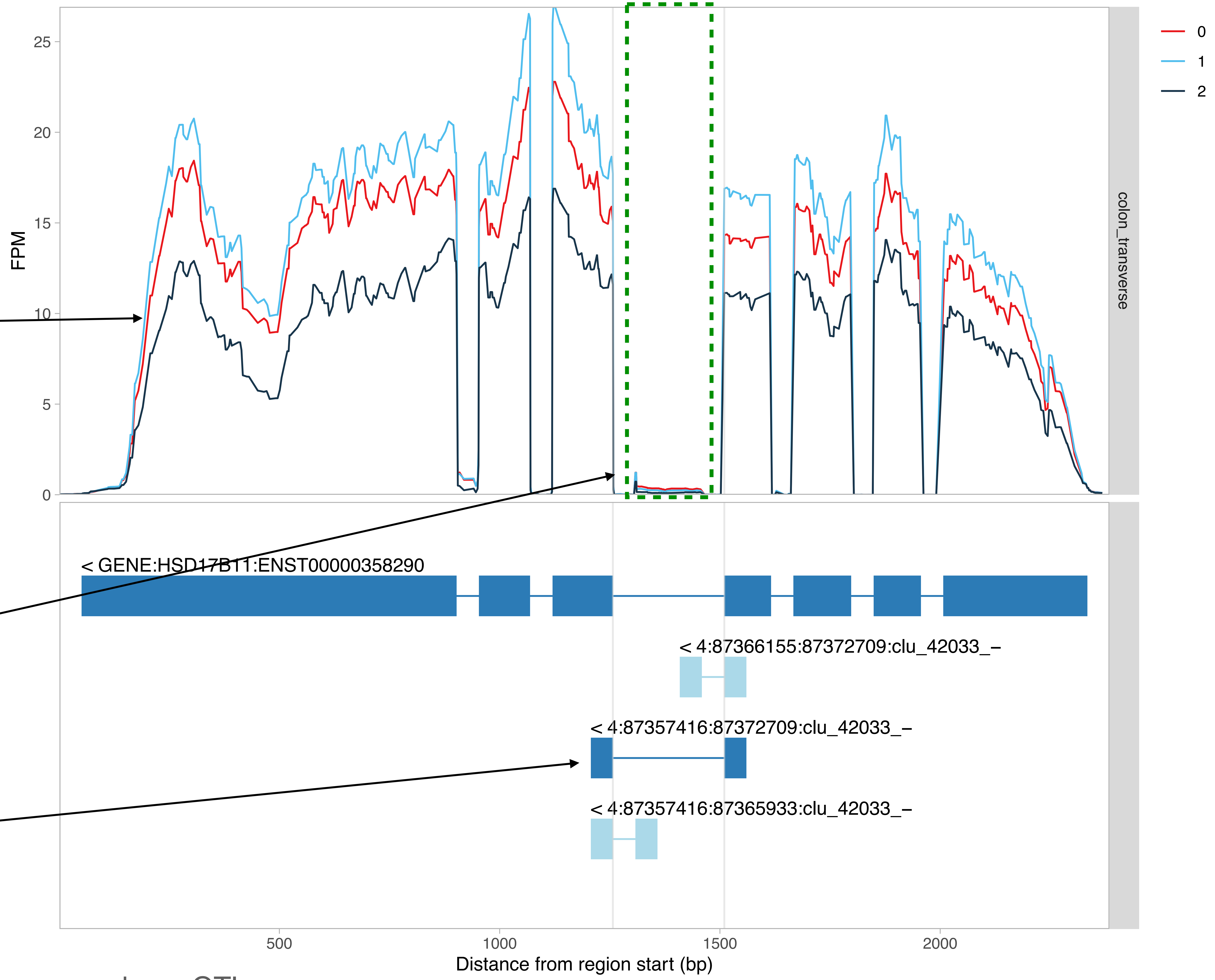

Decision: likely primary eQTL that manifests as a secondary sQTL

Examples of reference mapping bias

UKBB.VitD_chr4_85880379-88880005

Characteristic genotype-dependent bulge in read coverage in the middle of the exon.

Overlapping transcript annotations suggest alternative promoter usage, but this is not consistent with read coverage signal across the rest of the gene body.

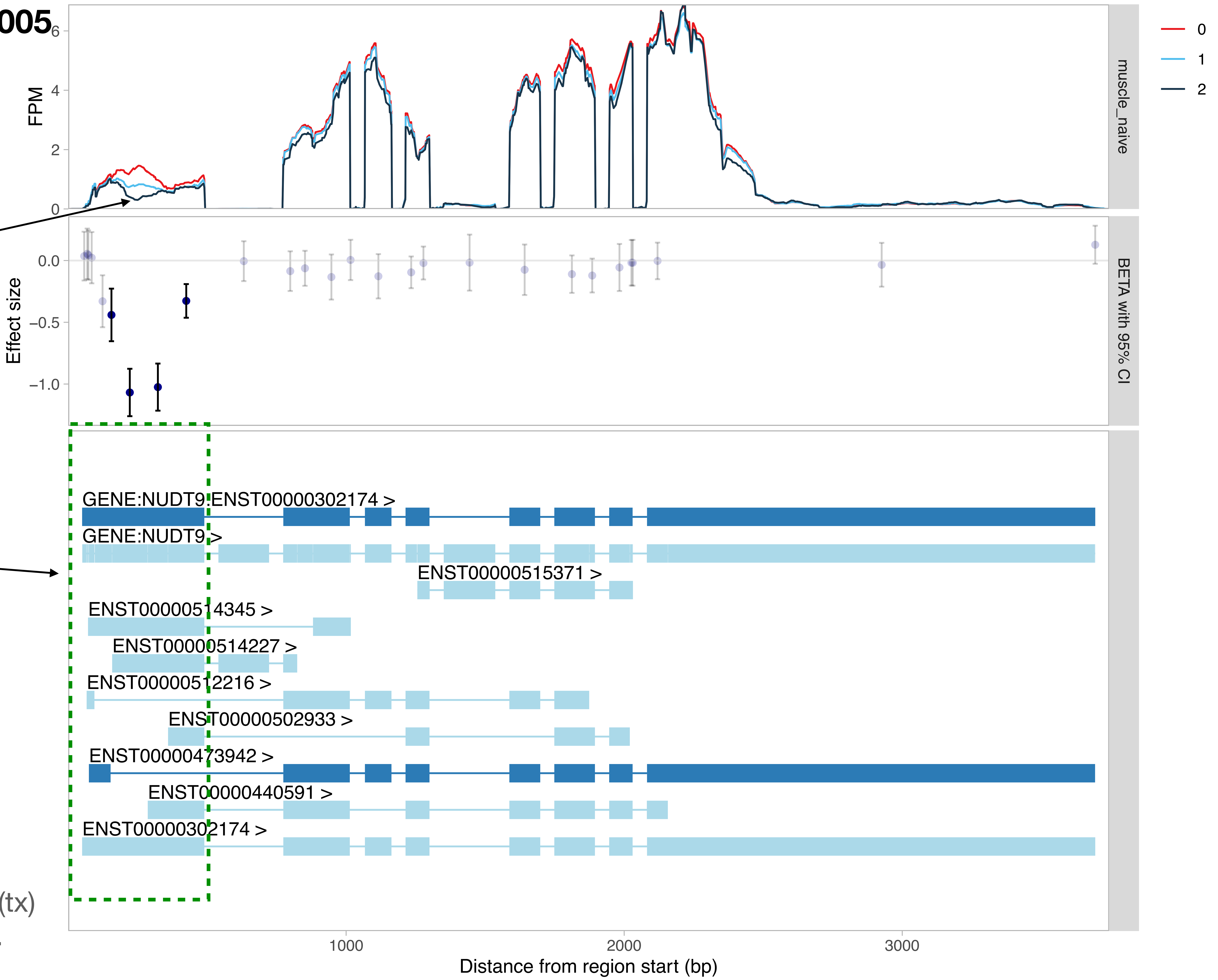

Decision: false positive transcript usage (tx) QTL driven by reference mapping bias.

UKBB.VitD_chr11_69846332-72949745

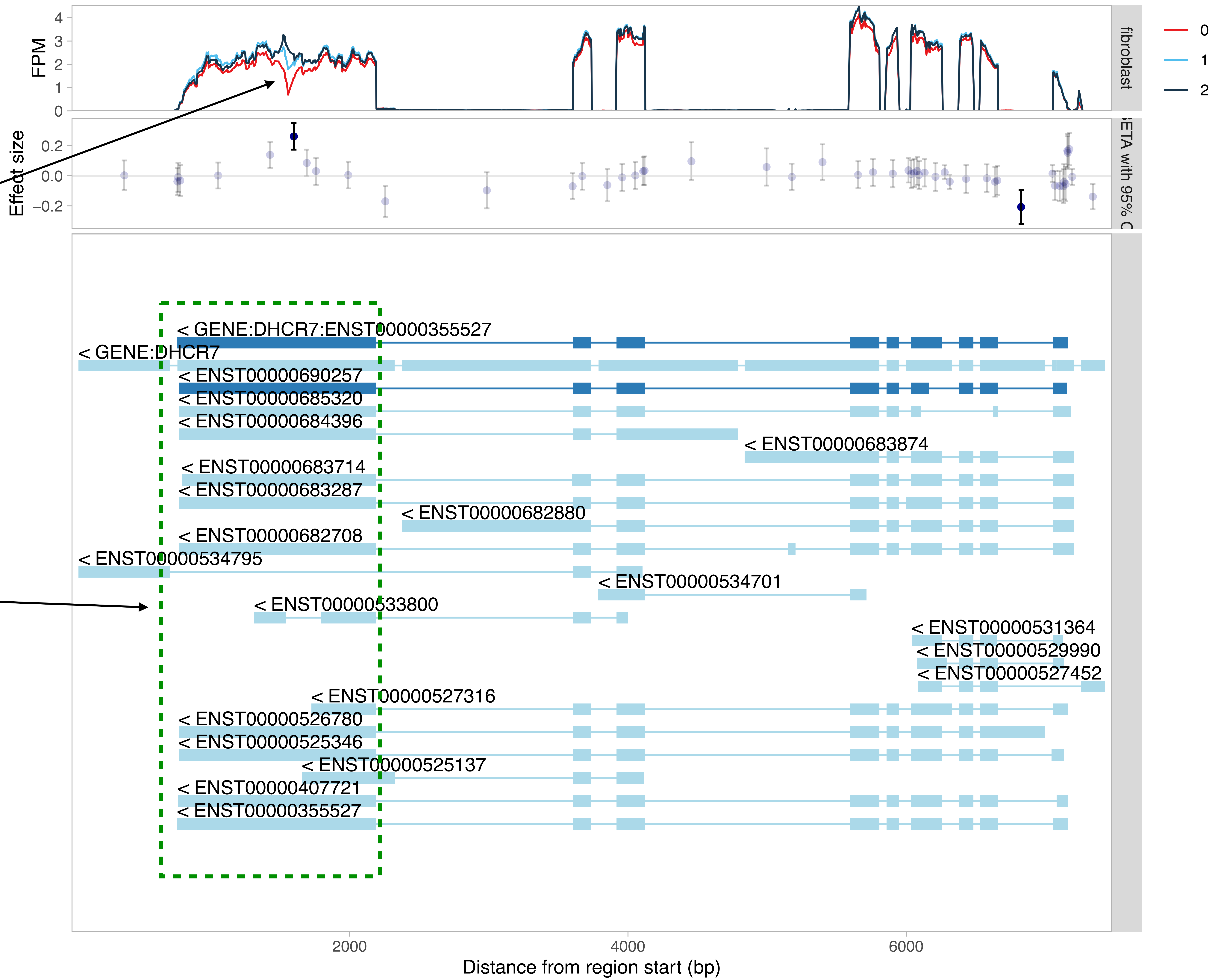

Characteristic genotype-dependent bulge in read coverage in the middle of the exon.

Overlapping transcript annotations suggest alternative last exon usage, but this is not consistent with read coverage signal across the rest of the gene body.

Decision: false positive transcript usage (tx) QTL driven by reference mapping bias.

UKBB.VitD_chr11_69846332-72949745

Characteristic genotype-dependent bulge in read coverage in the middle of the exon.

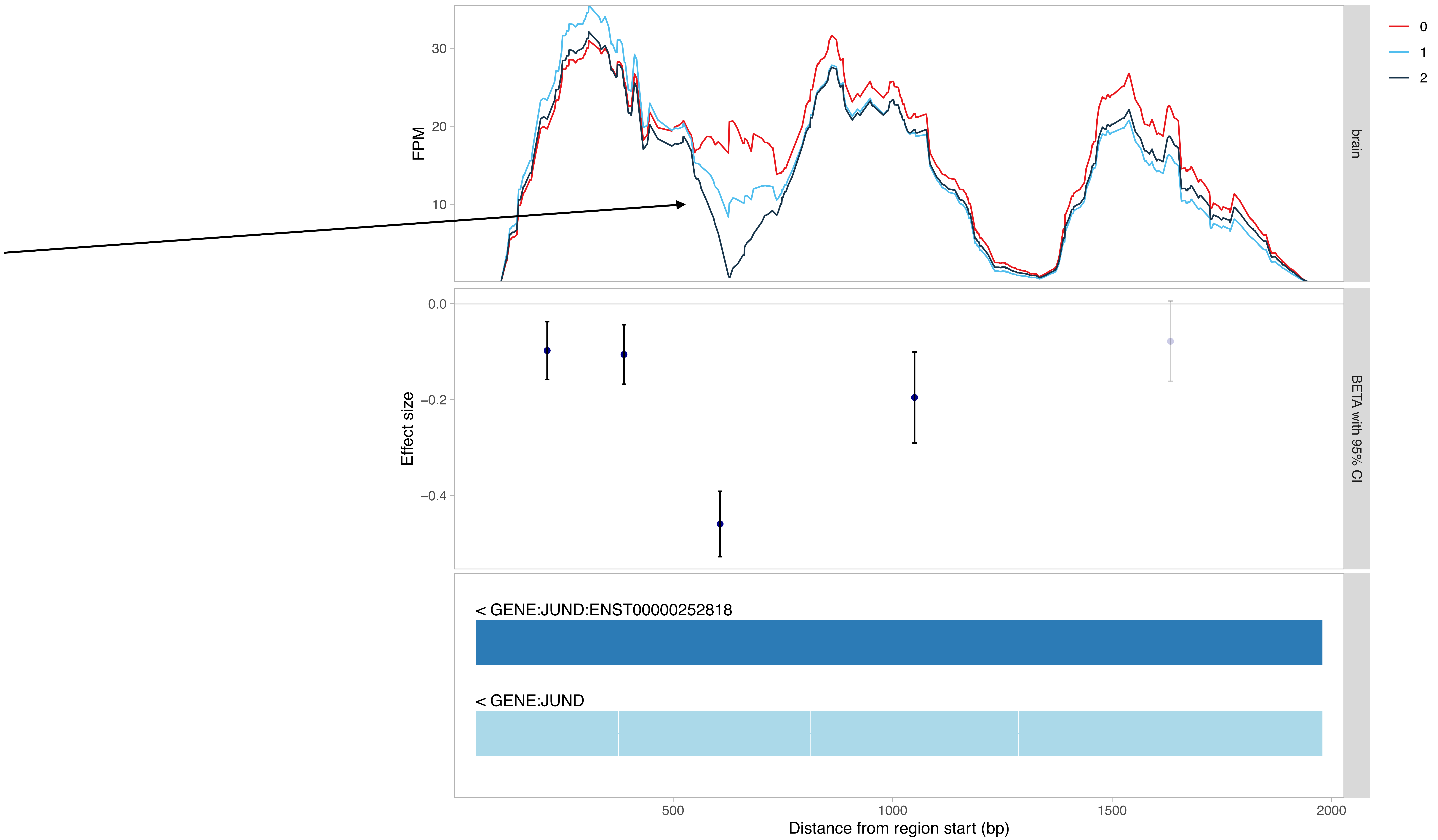

Decision: This seems to be a rare case where reference mapping bias leads to the detection of a false positive eQTL.
