# Supplementary material for: eQTL Catalogue 2023: New datasets, X chromosome QTLs, and improved detection and visualisation of transcript-level QTLs
Source: PLoS Genet. 2023 Sep 18;19(9):e1010932. doi: 10.1371/journal.pgen.1010932 (PMC10538656; doi:10.1371/journal.pgen.1010932)
Supplement: Attachment — Submitted filename: Response to Reviewers.pdf [file pgen.1010932.s006.pdf]

Response to reviewers

Reviewer #1: The authors present an update to the eQTL Catalogue, including new studies, inclusion of X chromosome eQTLs, as well as a new approach to discovery of expression/splicing QTLs that helps to identify most relevant signals within a gene, and de-prioritize other, correlated signals which may be less likely to be driving downstream phenotypes of interest. The resource, methods, and discussion are valuable for researchers in the fields of QTL discovery, and for QTL-GWAS colocalization efforts, with an emphasis on quality assessment via visual inspection of expression/splicing information within loci.

Thank you for the kind words.

Comments:

1) The authors mention that the various transcript-level association statistics are correlated and so have a large amount of redundancy that can be addressed with fine-mapping-based filtering. For a subset of genes, could the authors provide example correlation matrices, just for the reader to have an extent of the correlation of features being considered.

We now provide a detailed dissection of three primary sQTL loci colocalising with Vitamin D GWAS (*CYP2R1*, *HAL* and *SDR42E1*) in the supplement (Figures H-M in S1 Text), including correlation matrices and regional association plots for the quantified molecular traits, as well as lead sQTL variant boxplots using absolute (TPM) and relative (transcript ratios) quantification results. These examples indicated that although quantified molecular traits are often only moderately correlated, the detected genetic association signals are still highly similar, thus justifying our decision to filter the results.

We now discuss these examples also in the Methods (page 14 lines 407-410):
We provided three specific examples for genes SDR42E1, HAL and CYP2R1 on Figures H-M in S1 Text to illustrate how keeping one 'tag' molecular trait for each gene is able to faithfully capture the transcript-level QTL signals detect at these loci while significantly reducing the size of the summary statistics files.

2) Also related to the fine-mapping-based filtering: "only retain the most strongly associated molecular trait", is described in the Methods as using the highest PIP among a connected component formed over credible set input. If some features tend to have higher PIP values (across the whole dataset) does this shift the filtering toward those features? Is this related to the underling p-value distribution for the features? This comment is not suggesting to change the underlying methods or analyses. I am just curious do the authors think that considering the ranks within feature, rather than the raw PIP, would provide a different filtering outcome (that label of the molecular trait would be different)? This matters mostly, it would appear, for downstream tallying of mechanisms.

For most transcript/txrevise/leafcutter event usage QTLs there are typically at least two or more significant molecular traits with opposite effect size direction (See examples on Figures H-M in S1 Text). If the usage of one transcript increases with each copy of the alternative allele (positive beta), then by definition, there has to be at least one other transcript that exhibits decrease in usage (negative beta). Sometimes there are multiple alternative transcripts/splice junctions and the genetic effect gets diluted between those (e.g. Fig I and M in S1 Text), but the principle still holds. Our filtering strategy aims to pick one of those transcripts as a 'tag' transcript to significantly reduce the size of the summary statistics files, simplify colocalisation analysis, and make it easier to enumerate the number of independent signals detected.

We agree that selecting the transcript with the maximal PIP value is slightly arbitrary, but we do not think this choice has a significant impact on our results. First, PIPs are only calculated for those variant and molecular trait pairs for which SuSiE detects a "pure" credible set, which in practice means that there needs to be a significant association in the region. Secondly, PIP is typically correlated with p-values, because smaller standard errors of the betas mean that SuSiE can more confidently identify the likely causal variants. It definitely does happen that the transcript with maximal PIP is not always the same as the one with minimal p-value, but they are typically both ranked at the top (see e.g. Fig K in S1 Text). Finally, for any given genetic association, which transcript is selected as the 'tag' transcript does not change the interpretation of mechanism, because for that, all of the alternative transcripts still need to be considered jointly (e.g. by looking at the QTL coverage plot).

3) Is the coverage in Figure 2A log scale and is this why the genotypes 0 and 1 are closer to each other than 1 and 2?

Thanks for noticing this! The coverage track on Fig 2A is not on log scale, but we think that the larger difference seen between 1 and 2 compared to between 0 and 1 is primarily driven by the fact that this genetic variant has low minor allele frequency (MAF = 2%) and in this particular dataset there is only one individual with homozygous alternative genotype. We have now modified Fig 2A to indicate the number of individuals carrying each genotype.

4) While the authors note that manual inspection was used to assign mechanisms to different types of molecular QTL, it would be helpful to visualize the implied rules. Could the authors provide a table or a Sankey diagram linking the molecular QTL combinations to the empirical functional consequences?

We have now added the following clarification to page 9 (lines 208-213) of the main text:
Our aim was to distinguish primary splicing and transcript-level QTLs, where the putative causal variant has a direct effect on promoter or splice junction choice, from likely secondary associations, where the change in promoter, transcript or splice junction usage is likely a consequence of large-effect eQTLs, perhaps by altering the kinetics or fidelity of splicing [33,34]. Although this process is inherently subjective and noisy, we have provided some examples in S2 Text to clarify the implicit decision rules that we used.

Finally, we have added Sankey diagrams to illustrate how the molecular QTLs detected by different quantification methods are classified into primary mechanisms (Fig C in S1 Text). We decided to do this separately for QTLs detected with each quantification method as there were too many possible combinations to consider (see Fig 3A).

5) Regarding the multiple target genes in 3C, are these ever resolvable by examining the number of signals per gene? E.g. if a gene has two colocalized signals that may be a better candidate than another gene with only one colocalized signal.

We indeed have some preliminary evidence suggesting that this might be the case, but unfortunately there is only a small number of Vitamin D GWAS loci in our analysis where multiple independent variants colocalise with the same gene across all quantification methods. These are: *ADAM15*, *POU2F3*, *NADSYN1*, *HAL*, *HERPUD1*, *SDR42E1*, *GC*, *HSD17B11/HSD17B13* (See S3 Table). Of these, *HSD17B11/HSD17B13*, *GC*, *SDR42E1*, and *HAL* agree with our manually prioritized causal gene and *HERPUD1* disagrees (prioritized gene *CETP*). However, larger studies with better defined gold standards are needed to clarify this further, so we refrain from discussing this in detail in this paper.

Reviewer #2: This study by Kerimov et al provides an update to the eQTL Catalogue to facilitate downstream characterization of the exact mechanism of action at each molecular QTL (regulating expression, splicing, promoter usage, alternative polyadenylation, etc). This task of characterization has been a challenging problem for a number of reasons (for example, the substantial overlap in QTLs detected for gene expression, exon expression, txrevise, leafcutter, and transcript usage). This study presents an approach to generate QTL coverage plots for genetic signals of molecular traits to overcome these challenges.

Although this study is potentially useful to the research community (such as in supporting the development of new colocalization methods that assume multiple independent causal genetic effects), I have some major concerns about the way the study presents its methodology and results.

We thank the reviewer for constructive feedback and we believe that the changes we have made have significantly improved the clarity of the manuscript.

1. The Title, Introduction and Results all appear to show the relevance of the approach for a generic molecular QTL. However, the abstract itself restricts itself to splicing. The focus of the paper is thus quite confusing.

To add to the confusion, the first results presented concern the number of eGenes for the X chromosome. While I commend the authors for including these results on a generally neglected part of the genome, there needs to be more (and better) contextualization of these findings. The primary aim of the study, after all, is the development of a "systematic visualisation of molecular QTLs" for the purpose of enhancing the identification of the variant mechanisms at GWAS loci.

We agree that the initial title and abstract of the manuscript were misleading and too much focussed on a single aspect of the work (splicing visualisations). We have now changed the title and also completely re-written the abstract to emphasize that we are presenting a general updated to the eQTL Catalogue resource with multiple new features (new datasets, X chromosome QTLs, novel visualizations) followed by a case study on Vitamin D. We hope that the new title and abstract have improved the clarity of the manuscript.

For reference, here are the updated title and the abstract:

eQTL Catalogue 2023: new datasets, X chromosome QTLs, and improved detection and visualisation of transcript-level QTLs

The eQTL Catalogue is an open database of uniformly processed human molecular quantitative trait loci (QTLs). We are continuously updating the resource to further increase its utility for interpreting genetic associations with complex traits. Over the past two years, we have increased the number of uniformly processed studies from 21 to 31 and added X chromosome QTLs for 19 compatible studies. We have also implemented Leafcutter to directly identify

splice-junction usage QTLs in all RNA sequencing datasets. Finally, to improve the interpretability of transcript-level QTLs, we have developed static QTL coverage plots that visualise the association between the genotype and average RNA sequencing read coverage in the region for all 1.7 million fine mapped associations. To illustrate the utility of these updates to the eQTL Catalogue, we performed colocalisation analysis between vitamin D levels in the UK Biobank and all molecular QTLs in the eQTL Catalogue. Although most GWAS loci colocalised both with eQTLs and transcript-level QTLs, we found that visual inspection could sometimes be used to distinguish primary splicing QTLs from those that appear to be secondary consequences of large-effect gene expression QTLs. While these visually confirmed primary splicing QTLs explain just 6/53 of the colocalising signals, they are significantly less pleiotropic than eQTLs and identify a prioritised causal gene in 4/6 cases. All our association summary statistics and QTL coverage plots are freely available at https://www.ebi.ac.uk/eqtl/.

2. Figure 1c shows that the newly added datasets since the previous release appear to be low sample size datasets. Therefore, rather than showing the number of eGenes from the previous release and from the new datasets, it would be important to show how many new eGenes were actually identified through inclusion of these new (low sample size) datasets. What was gained from their inclusion?

We have now included an analysis where we quantify the number of novel eGenes detected with each additional dataset added to the eQTL Catalogue. We find that the increase in number of completely novel eGenes in datasets added between releases 3 and 6 is relatively small (1.8%) and primarily driven by one dataset with the largest sample size (CommonMind prefrontal cortex) (Fig B in S1 Text). However, this might reflect saturation as the total number of unique eGenes is now at 27,067, covering a large majority of expressed coding and non-coding genes. To gain further resolution, we also looked at the number of unique confidently fine-mapped gene-variant pairs (posterior inclusion probability > 0.95) and found that these increased by 4%, suggesting that novel eQTLs can still be discovered when well-powered studies are available.

We have now added the following section to the results (page 5 lines 145-150):
"For gene expression QTLs, we further quantified the number of novel associations detected in release 6 relative to release 3. The number of unique genes with at least one eQTL ('eGenes') increased by 1.8% whereas the number of confidently fine mapped eQTL gene-variant pairs (posterior inclusion probability > 0.8) increased by 4% (Fig B in S1 Text). Most of this increase was driven by the CommonMind [16] prefrontal cortex dataset that had the largest sample size (n = 586)."

3. Finally, without a precise description of the statistical methodology for classifying a signal (into an expression QTL, promoter usage QTL, splicing QTL, etc.) -- given the potentially substantial sharing of significantly colocalized signals, as in the example of vitamin D (figure 3) -- the value/quality of the "visualization" approach and the resulting resource of QTL coverage plots is really quite challenging to assess. I worry that without a rigorous statistical framework to guide interpretation, the resource will have very limited impact.

We have now added the following clarification to page 9 (lines 208-213) of the main text:
Our aim was to distinguish primary splicing and transcript-level QTLs, where the putative causal variant has a direct effect on promoter or splice junction choice, from likely secondary associations, where the change in promoter, transcript or splice junction usage is likely a consequence of large-effect eQTLs, perhaps by altering the kinetics or fidelity of splicing [33,34]. Although this process is inherently subjective and noisy, we have provided some examples in S2 Text to clarify the implicit decision rules that we used.

We completely agree that trying to assign mechanisms to molecular QTLs in this way is inherently noisy and subject to experimenter bias in ways that is difficult to control for. In the future, we believe that assignment of mechanism could be done more accurately with integrative machine learning models that take into account the sequence context and annotations of the associated variants. However, we believe that our visualizations are still useful for two main purposes:
1. Interpreting molecular QTLs that colocalise with complex traits at specific loci where the underlying signal is strong enough for visual classification (e.g. *CYP2R1* and *SDR42E1* examples on Fig H and J in S1 Text).
2. Assembling training and validation datasets to train the integrative machine learning models mentioned above.
